# Inflammatory Cytokine Signaling during Development of Pancreatic and Prostate Cancers

**DOI:** 10.1155/2017/7979637

**Published:** 2017-12-12

**Authors:** Geou-Yarh Liou

**Affiliations:** Center for Cancer Research & Therapeutic Development and Department of Biological Sciences, Clark Atlanta University, 223 James P. Brawley Drive SW, Atlanta, GA 30314, USA

## Abstract

Inflammation is essential for many diseases including cancer. Activation and recruitment of immune cells during inflammation result in a cytokine- and chemokine-enriched cell environment, which affects cancer development. Since each type of cancer has its unique tumor environment, effects of cytokines from different sources such as tumor-infiltrating immune cells, stromal cells, endothelial cells, and cancer cells on cancer development can be quite complex. In this review, how immune cells contribute to tumorigenesis of pancreatic and prostate cancers through their secreted cytokines is discussed. In addition, the cytokine signaling that tumor cells of pancreatic and prostate cancers utilize to benefit their own survival is delineated.

## 1. Introduction

Cancers develop because of a dysfunctional immune system of the body, which is unable to detect or eliminate precancerous cells at an early stage. Hijacking the immune system, which eliminates pathogens and unwanted cells such as senescent, damaged, or immature cells under normal physiological conditions, is a very common strategy that cancers utilize for benefitting their long-term growth and survival against locally limited resources, for example, less oxygen in solid tumor tissues. To overcome this shortage of resource, cancer cells express several cytokines, growth factors, and receptors for cytokines and growth factors to become independent of the mitogens that are supplied other than themselves. Another mechanism, which cancer cells utilize, is to recruit immune cells such as macrophages, neutrophils, B cells, and so on, and these tumor-infiltrating immune cells interact with cancer cells, cancer-associated fibroblasts, and themselves. Meanwhile, tumor-associated immune cells secrete certain cytokines, chemokines, and proteases, for example, TGF*β* to dampen T cells, natural killer (NK) cells, and dendritic cells; all of which are engaged in eradicating cancer cells. The indirect effects of immune cells among themselves also create a tumor-favorable outcome. For example, IL-10 secreted by *γδ* Treg cells diminished the cytotoxic activity of CD8^+^ T cells and NK cells, resulting in tumor growth [1]. It is certainly complex that how different types of tumor-infiltrating immune cells affect their biological functions among each other through self-secreted cytokines and chemokines, leading to specific and unique tumor environments in different types of cancer. Moreover, the neighboring cell types around cancer cells also contribute to distinct tumor environments in different types of cancer. For example, breast cancer is neighbored by adipose tissue; pancreatic cancer is accompanied by massive desmoplasia. Given that the same cytokine can result in opposite biological effects in different types of cancer, that is, tumor promoting versus tumor inhibiting due to their unique tumor environment, it is important to comprehensively review cytokine signaling and its functions during cancer development in each type of cancer for shedding light on cancer therapies by altering the tumor environment. In this review, I will mainly focus on cytokine signaling of tumor-facilitated immune cells and of cancer cells that lead to tumor initiation, progression, and metastasis of pancreatic cancer and prostate cancer.

## 2. Pancreatic and Prostate Cancers

Pancreatic cancer is the most lethal type of cancer with an approximately 7% 5-year survival rate and is projected to become the 2nd leading cause of all cancer-related death by 2030. Pancreatic ductal adenocarcinoma (PDAC) represents over 90% of pancreatic cancer cases. Oncogenic Kras mutations are present in almost all PDAC patients and are required to initiate and develop PDAC. Another unique feature of PDAC is severe desmoplasia/fibrosis, which occurs at the very early stage of the disease. Pancreatic stellate cells, fibroblasts, and enriched extracellular matrix (ECM) components from desmoplasia orchestrate with other types of cells including immune cells and endothelial cells to promote pancreatic cancer growth and survival.

Prostate cancer is the most common type of cancer in men. In addition, it is also the 2nd leading cause of cancer fatality in men. Unlike PDAC, which is a genetic disorder of oncogenic Kras, key genetic mutations lead to prostate cancer remain unclear. Although several genetically engineered mouse models are generated for studying prostate cancer initiation, development, and dissemination *in vivo*, none of them fully recapitulates this disease in humans. Most *in vivo* studies, especially for testing potential therapeutic drugs on prostate cancer, mainly rely on xenograft mouse models using human prostate cancer cell lines or patient tissue samples. It restrains the advancements of how these drugs also affect immune cells and stromal cells, which can contribute to drug efficacy during cancer therapy.

Although pancreatic and prostate cancers are quite different and face different challenges for cancer therapies, the patients of these two cancers share certain features among other cancer patients which include (1) late-onset diseases (in patients' late 60), (2) a gender preference which males rather than females are prone to the diseases, and (3) an ethnic preference which African Americans rather than other racial groups are more susceptible to these cancers. Of note, both cancer types are tightly associated with old age, implicating a pivotal role of aging-related inflammation in these two types of cancer. Herein, an overview of how the cytokine signaling from immune cells and cancer cells affects tumorigenesis of the pancreas and the prostate is provided below.

## 3. Cytokine Signaling from Immune Cells for Modulating Tumorigenesis

**Figure 1:**
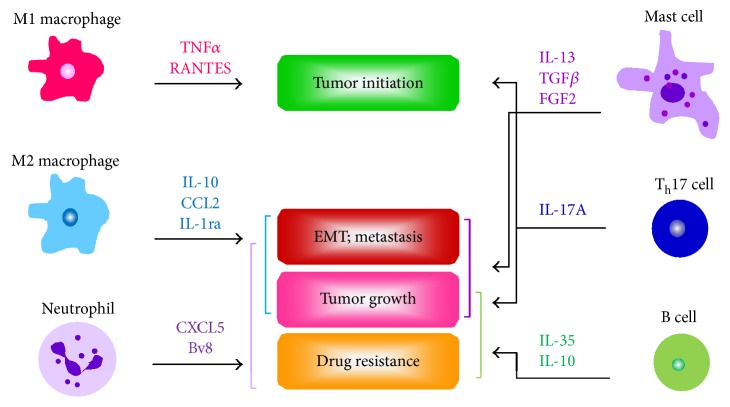
Effects of the cytokines secreted by different types of immune cells on tumor initiation, progression, and dissemination. Cytokines secreted by macrophages, neutrophils, mast cells, T_h_17 cells, and B cells directly lead to initiation and development of pancreatic and prostate cancers. IL: interleukin; RANTES: regulated on activation, normal T cell expressed and secreted; TGF*β*: transforming growth factor *β*; FGF2: fibroblast growth factor 2; EMT: epithelial mesenchymal transition.

The immune system is consisted of many types of immune cells, organs, and antibodies to protect and defend our body. Immune cells can be majorly categorized to phagocytes and lymphocytes which secrete cytokines and chemokines to alter a local cell microenvironment. It has been demonstrated that several cytokines from both phagocytes and lymphocytes potentiate tumor initiation, proliferation, and metastasis of pancreatic and prostate cancers (Figure 1). How these cytokines secreted by different types of immune cells to directly influence cancer cells or indirectly affect nearby immune cells during cancer development are described below in details.

### 3.1. Cytokines Secreted by Phagocytes and Their Functions

Phagocytes include macrophages, neutrophils, mast cells, and dendritic cells and are capable of ingesting microorganisms, cellular debris, and foreign particles. A large and convincing body of evidence demonstrates the increased infiltrating macrophages and their role in cancer progression and dissemination of PDAC and prostate adenocarcinoma. IL-6 from infiltrating macrophages in the pancreata of PDAC transgenic mice activated transcription factor STAT3 in pancreatic tumor cells, promoting tumor development [2]. Macrophages are categorized to M1 and M2 subtypes, which are also known as classically activated (M1) and alternatively activated (M2) according to their activations (see details in review [3]). M1 macrophages are activated by lipopolysaccharide (LPS), interferon gamma (IFN*γ*), virus, and so on and participate in killing pathogens and clearing up dying cells to protect the host, known as inflammation. During this process, M1 macrophages secrete inflammatory cytokines and generate nitric oxide (NO). However, M2 macrophages stimulated by interleukin-4 (IL-4), IL-13, IL-10, and so on produce extracellular matrix components, angiogenic and chemotactic factors, and IL-10. M2 macrophages are engaged in wound healing, tissue remodeling, allergy, and immunoregulation. Based on the characters of M1 and M2 macrophages described above, several markers such as iNOS, CD38 (for M1 macrophages), Egr2, and CD163 (for M2 macrophages) have been utilized for studying their functions on cancer initiation, development, and dissemination [4–6]. Increased numbers of M2-polarized tumor-associated macrophages (TAM) were reported to correlate with a poor prognosis of PDAC [7, 8]. Of note, M1 proinflammatory macrophages, which generally are thought to be antitumor, have been demonstrated to initiate PDAC development [9–11]. TNF*α* and RANTES secreted by M1 macrophages upregulate expressions and activities of matrix metalloproteinase 9 (MMP9) of pancreatic acini, resulting in transdifferentiating these cells to a progenitor duct-like phenotype, which later can further become PDAC cells. This piece of evidence also provides a plausible and common scenery of how inflammation-related conditions such as pancreatitis, obesity, diabetes, aging, and so on increase the risk of getting PDAC.

In a pdx1^cre^:Kras^G12D^:Trp53^R172H^ (KPC) transgenic mouse model that recapitulates human metastatic PDAC, depletion of macrophages by pharmacological compound clodronate liposomes at an early stage of PDAC reduced metastatic cancer cells in the liver and lungs [12]. M2-polarized macrophages secreted high levels of CCL2 and IL-1ra around preinvasive pancreatic intraepithelial neoplasia (PanIN) lesions *in vivo*, resulting in elevated proliferation of PanIN cells through enhanced ERK signaling [13]. In addition, prevention of macrophage polarization to a M2 subtype by an IL-13 neutralizing antibody diminished fibrosis, which is associated with pancreatic tumor proliferation and progression. In a coculture system, in addition to elevated cancer cell proliferation and migration, IL-4-polarized M2 macrophages also induced epithelial-mesenchymal transition (EMT) in human pancreatic cancer cell lines [14]. Furthermore, depletion of toll-like receptor 4 (TLR4) or neutralization of IL-10 in M2 macrophages blocked the EMT ability of cancer cells. Very recently, it has been demonstrated that inflammasome component protein NLRP3 expressed by M2 macrophages (CD206^+^MHCII^−^) is essential for their proliferation in PDAC [15]. In addition, these NLRP3^+^ macrophages also resulted in increased populations of T_h_17 and Treg cells and an inactivation of cytotoxic CD8^+^ T cells. Interference with inflammasome by targeting its component proteins through pharmacological compounds or NLRP3 knockout reduced PDAC formation *in vivo*.

Accumulating evidence indicated an increased recruitment of M2 immunosuppressive macrophages in prostate cancer, associating with cancer growth, metastasis, and drug resistance, thus leading to worse outcomes for prostate cancer patients [16–20]. However, to date, research studies in prostate cancer field majorly are focused on how prostate cancer affects macrophage polarization and infiltration due to several discrepant clinical data of inflammation/macrophages in prostate cancer, which may be due to varied markers of different immune cell types and quality of specimens.

Several lines of evidence have demonstrated involvement of neutrophils in cancer initiation, progression, and dissemination. These processes are mediated by neutrophil-derived products including cytokine, chemokines, proteases, reactive oxygen species (ROS), and so on (see detailed reviews in [21, 22]). However, the detailed mechanisms of how these tumor-associated neutrophils (TAN) promote cancer development and metastasis, especially in varied tumor environments of different types of cancer, remain unclear. A neutrophil-expressed glycoprotein lipocalin 2, known for combatting bacterial infection, was present in preinvasive PanIN lesions and PDAC and was suggested as a marker for early diagnosis of PDAC [23]. In addition, the ratio of neutrophil to lymphocyte found in the peripheral blood was correlated to the overall survival of PDAC patients [24, 25]. TAN secretes several cytokines and chemokines such as CXCL5, TGF*β*, and TNF during tumor development and metastasis. In transgenic KPC mice of PDAC, high levels of CXCL5 in the tumor stroma were detected [26]. Moreover, knockout of CXCL5 receptor CXCR2 in PDAC cells dramatically blocked liver metastases. In a xenograft mouse model of human pancreatic cancer AsPC-1 cells, administering an inhibitor of chemokine Bv8, which is secreted by TAN, reduced tumor size and enhanced gemcitabine-induced cytotoxicity of cancer cells [27].

Mast cells participate in many physiological processes such as immune tolerance, angiogenesis, wound healing, and defense against pathogens. Dysregulation of mast cell activation associates with many disorders including cancer. Increased infiltration of mast cells was reported in PDAC patient samples and correlates with a high grade of tumor as well as a poor prognosis [28, 29]. Implantation of murine cancer Pan02 cells into the pancreas tissues of mast cell-deficient mice failed to develop PADC *in vivo* [30]. It has been shown that neutralization of IL-13 secreted by mast cells hindered proliferation of human pancreatic stellate cells (PSC) [31]. Interestingly, blockade of IL-13 also decreased TGF*β* expression and activation of Smad2 in PSC, suggesting that TGF*β* signaling as downstream of IL-13 promotes PSC proliferation.

Similar to that in PDAC patients, more mast cells were present in prostate adenocarcinoma tissues and further increased their recruitment to castrate-resistant prostate tumors [32]. Less mast cells present within prostate tumors may indicate a high recurrence rate of the disease [33]. In an orthotopic rat model of prostate cancer, coinjection of mast cells with cancer AT-1 cells increased tumor growth [32]. Moreover, increased mRNA levels and protein expressions of fibroblast growth factor 2 (FGF2) were detected in mast cell-infiltrating prostate tumors. In a xenograft mouse model, coimplantation of human prostate cancer cells with mast cells enhanced tumor metastasis to the diaphragm [34]. In addition, cultured mast cells downregulated mRNA levels of androgen receptor of human prostate cancer cell lines, leading to increased invasiveness and migration ability of cancer cells.

### 3.2. Cytokines Secreted by Lymphocytes and Their Functions

Lymphocytes include T cells, B cells, and natural killer (NK) cells. Dysfunctional T cells generated through different molecular and cellular mechanisms such as T cell anergy, exhaustion, and senescence allow tumor growth by escaping immune surveillance. In addition, presence of regulatory T cells (Treg), which suppress self-activation of T cells, also contributes to immunosuppressive tumor microenvironments, blocking antitumor immune responses, and subsequent cancer progression. In tumor tissue samples of PDAC patients, more CD4^+^CD25^+^Foxp3^+^ Treg cells were detected and linked to a poor prognosis [35, 36]. This increase in Treg cells also correlates with less cytotoxic CD8^+^ T cells and CD4^+^ T helper cells in PDAC and precancerous PanIN lesions. Reprogramming of Treg cells by a CD25 neutralizing antibody daclizumab results in an increase in CD56^bright^ natural killer cells and functional T cells such as cytotoxic CD8^+^ T cells and CD4^+^ T helper cells in a small clinical trial of metastatic breast cancer patients who receive a cancer vaccine [37]. Results from a heterotopic mouse model of PDAC using nonmetastatic murine Pan02 cancer cells and an orthotopic xenograft mouse model using metastatic human Panc-1 cancer cells revealed that pancreatic cancer expresses CCL5 to recruit CD4^+^Foxp3^+^ Treg cells, which have high levels of CCR5 [38, 39]. Interruption of this interaction between CCL5 and CCR5 by knocking down CCL5 in cancer cells that were used for cancer cell transplantation in mice impedes cancer growth. This suppression effect can also be achieved by administering a CCR5 inhibitor TAK-779- or a CCL5-neutralizing antibody in mice. IL-10 secreted by CD4^+^Foxp3^+^ Treg cells renders T cell anergy (unresponsive to IL-10 restimulations) (see review in [40, 41]). IL-10 from Treg can also prevent T cell expansion by directly suppressing IL-2 production in T cells. In addition to induction of IL-10 expression in Foxp3^+^ Treg cells, TGF*β* from CD4^+^Foxp3^+^ Treg cells and pancreatic cancer also stimulates transcription factor Foxp3 expression in Foxp3^−^ naïve T cells, leading to differentiation of Foxp3^+^ Treg cells [42, 43]. Whether targeting Treg cells, for example, through CD25 blockade or Treg-secreted cytokines as mentioned previously is sufficient to overcome immune resistance of pancreatic cancer *in vivo* remains to be evaluated. In prostate cancer, a high density of Treg cells was detected in cancer tissues and associated with an advanced tumor stage as well as a low survival rate [44, 45]. In prostate cancer with bone metastasis, Treg cells expressing high levels of CXCR4 were recruited to bone marrow through an interaction with CXCL12, which is enriched in the bone marrow [46]. Meanwhile, the Treg cells in the bone marrow upregulated RANKL expression to increase their proliferation, which is mediated by RNAK^+^ dendritic cells.

T helper 17 (T_h_17) cells are T helper cells producing IL-17 and involved in autoimmune and inflammatory disorders. In response to different stimuli including IL-6, TGF*β*, CCL20, IL-23, IL-1*β*, and so on, especially under cancer microenvironments, Th17 cells can mediate tumor regression or tumor promotion [47–49]. In a PDAC heterograft mouse model, increased infiltration of Th17 cells was observed in IL-6-expressed tumors that cause high mortality in mice [50]. In a transgenic mouse model of PDAC, oncogenic Kras^G12D^ induced IL17A-expressed T_h_17 cells, leading to tumor initiation and progression of precancerous PanIN lesions [48]. This initiation and promotion effects by T_h_17 cells are mediated through their interaction with tumor-initiating cells and PanIN cells, both expressing IL-17 receptor A (IL-17RA). In addition, manipulation of IL-17A by overexpression of IL-17A in the pancreas or knockout of IL-17A in hematopoietic cells interfered initiation and development of pancreatic tumor *in vivo*. In a xenograft mouse model of human pancreatic cancer CFPAC-1 cells, depletion of IL-17RB impedes cancer growth and metastasis to the liver and lungs [51]. The effect of IL-17RB on PDAC proliferation and dissemination was mediated by upregulation of CCL20, CXCL1, and IL-8 cytokines through ERK signaling in PDAC cancer cells. In prostate cancer, more T_h_17 cells and higher levels of its secreted cytokine IL-17A are linked to a worse outcome of patients [52, 53]. Knockout of IL-17RC, a receptor for IL-17A, decreased invasive prostate tumor growth of PTEN^−/−^ transgenic mice through downregulation of MMP7 and increased expressions of MMP inhibitors TIMP1, 2, and 4 [54]. Interference with T_h_17 cells by administration of SR1001, a small molecule inhibitor targeting T_h_17 transcription factors ROR*γ*t and ROR*α*, or with T_h_17 cell-secreted IL17 by an IL-17-neutralizing antibody, results in reduced cancer proliferation, angiogenesis, and inflammation of PTEN^−/−^ mouse prostate tissues [55].

Microarray study using human samples of PDAC demonstrated increased mRNA expressions of CD20, a marker for B cells [56], suggesting an association of B cells with PDAC development. Infiltration of protumorigenic B cells is associated with hypoxia and fibroblast-enriched stroma [57, 58]. Depletion of B cells, such as use of a CD20 neutralizing antibody or B cell-deficient mice, significantly delays the growth of PDAC and its precursor pancreatic intraepithelial neoplasia (PanIN) in transgenic and orthotopic mouse models [56–58]. Furthermore, reconstitution of functional B cells obtained from wildtype mice through adoptive transfer method rescued pancreatic tumor growth. Among these protumorigenic B cells, which are CD1d^hi^CD5^+^, increased levels of IL-35 were observed and speculated to be responsible for pancreatic tumor growth [58].

Although an increased density of B cell within prostate tumors has been reported in prostate cancer patient samples [59, 60], so far, only a handful publications indicate the function of tumor-infiltrating B cells on tumorigenesis of prostate cancer. In castration-resistant prostate cancer from the xenograft mice, release of IL-1*α* from the necrotic primary cancer cells results in CXCL3 secretion, which, in turn, recruits B cells. These tumor-infiltrating B cells produce lymphotoxin to stimulate cancer growth under hormone-independent conditions [61]. In Oxaliplatin-resistant prostate cancers of TRAMP transgenic mice, increased numbers of B lymphocytes were detected [59]. These infiltrating B cells expressed high levels of IL-10, IgA, and programmed death ligand 1 (PD-L1), leading to the development of oxaliplatin drug resistance over time. Moreover, ablation of IL-10, PD-L1, or B cells reinstated prostate cancer sensitivity to Oxaliplatin-induced cell death.

## 4. Cytokine Signaling from Tumor Cells for Modulating Tumor Growth and Proliferation

**Figure 2:**
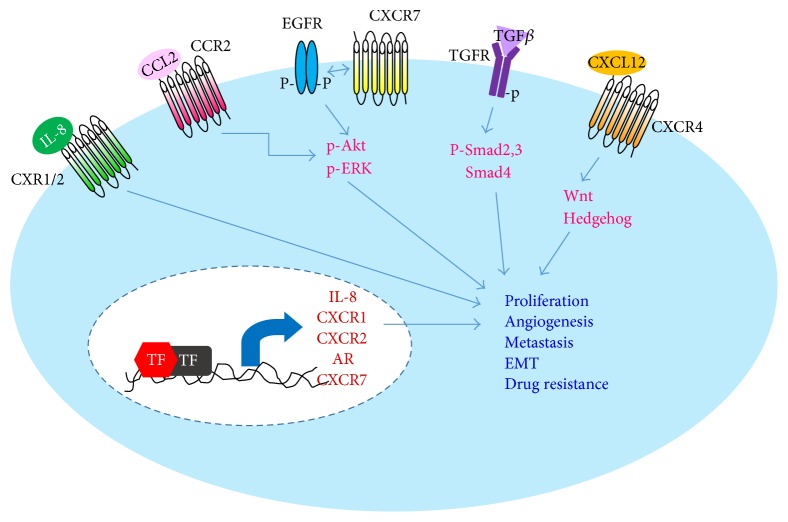
Cytokine signaling of cancer cells on modulation of cell proliferation, angiogenesis, metastasis, and drug resistance. Cytokine signaling pathways utilized by pancreatic and prostatic tumor cells through an autocrine-signaling mechanism to elevate their own growth, angiogenesis, and drug resistance. In addition, upregulation of these signaling molecules also renders tumor cells a more mesenchymal-like phenotype, which in turn, promotes metastasis. TF: transcription factor; IL-8: interleukin-8; AR: androgen receptor; EMT: epithelial mesenchymal transition; p: phosphorylated.

A large and still continuously increasing body of evidence demonstrated that cancer cells upregulate expressions of numerous cytokines to benefit their own survival. This is achieved by modulating surrounding cells to create a tumor-promoting environment. For example, pancreatic tumor cells expressed cytokine IL-13 to repolarize the nearby macrophages to a tumor-promoting/M2 subtype [13]. In addition, cancer cells also express receptors for the upregulated cytokines to persistently support their own growth (known as autocrine-signaling mechanism, see Figure 2) and become more independent on limited exogenous growth factors as well as mitogens. Insights into these cytokine-signaling pathways of cancer cells could shed light on developing a better cancer therapy for PDAC and castrate-resistant prostate adenocarcinoma.

Increased levels of mRNA and protein of IL-8 were detected in human pancreatic cancer cell lines and PDAC patient tissues ([62, 63]; see reviews in [64]). Elevated expression of IL-8 in PDAC tumor under hypoxic conditions associated with cancer metastatic ability in xenograft mice [65]. In addition, downregulation of IL-8 by expressing a specific antisense oligonucleotide against IL-8 in PDAC cells before implantating in mouse pancreas hindered tumor vascularization, leading to smaller tumors. Upregulation of IL-8 in PDAC is mediated by transcription factors AP-1 and NF-*κ*B; both positively modulate IL-8 promoter upon oncogenic Kras mutation [66]. Besides, administration of an IL-8-neutralizing antibody, which blocks IL-8 signaling, in xenografted mice resulted in elevated tumor necrosis and decreased angiogenesis without affecting tumor proliferation. In addition to IL-8, its receptors CXCR1 and CXCR2 were also reported in human PDAC Capan-1 cells to regulate cancer cell growth, migration, invasion, and its stem cell-like features [62, 63]. In human androgen-independent prostate adenocarcinoma, stronger expressions of IL-8 were present and correlated with advanced stages of the disease [67, 68]. Depletion of IL-8 by shRNA technique in cultured prostate cancer cells, which are derived from human castrate-resistant prostate adenocarcinoma, led to reduced cell proliferation and migration and enhanced cytotoxicity in response to chemotherapeutic drugs such as docetaxel [69]. Several signaling pathways involved in potentiating cell proliferation of prostate cancer by IL-8 have been delineated. Increased IL-8 of prostate cancer cells signals through CXCR1 and CXCR2 to induce transcription of androgen receptor (AR) and to activate ERK and Akt, in turn, promoting cancer growth and survival [68, 70]. In addition, IL-8 of the prostate cancer cells also upregulated EGFR-ERK signaling through elevating CXCR7 transcripts and protein levels [71]. However, the mitogenic function of CXCR7 in prostate cancer is ligand independent.

Higher expressions of TGF*β* have been detected in human PDAC tissue samples and associated with a better prognosis of PDAC patients [72–74]. In human pancreatic cancer Panc-1 cells, results from knockdown of TGF*β* signaling mediators Smad2 and Smad3 demonstrated an opposite function of Smad2 versus Smad3 on TGF*β*-induced cell migration [75]. Silencing Smad4 in Panc-1 cells impeded epithelial-to-mesenchymal transition ability of cells (increased E-cadherin; decreased N-cadherin and vimentin), and this alteration of EMT is mediated by loss of nestin, a protein expressed in stem cells during development [76]. In the KPC transgenic mouse model of PDAC, cancer precursors, which are acinar-to-ductal metaplasia (ADM) and PanIN, and PDAC expressed higher levels of activated Smad2 [77]. Moreover, suppression of TGF*β* by a TGF*β*-neutralizing antibody in these mice expedited PDAC proliferation and malignant progression and caused death, suggesting an inhibitory effect of TGF*β* during PDAC growth and development. In addition to stromal cells as well as the cells of benign prostatic hyperplasia (BPH) and of prostatic intraepithelial neoplasia (PIN), increased mRNA and protein levels of both TGF*β* and its receptors were detected in cancer cells of human and rat prostate adenocarcinoma tissues [78, 79]. In an allotransplantation model of rat prostate cancer cells that overexpressed TGF*β*, these tumors grew faster and metastasized to the lungs and lymph nodes. Treating these animals with a TGF*β*-neutralizing antibody rescued the phenotype [80]. Moreover, when the TGF*β*-expressed cancer cells were cultured *in vitro* in 2D, their proliferation were inhibited as numerous cases reported in cultured human prostate cancer cell lines. This result suggested the importance of cell environment regarding the impact of TGF*β* expressed by cancer cells on their own proliferation and malignancy.

Increased CCL2 expression was present in PDAC tissues and a portion of human PDAC cancer cell lines [13, 81]. Moreover, no CCR2, a receptor for CCL2, was detected in the tested human CCL2-expressed PDAC cell lines. This may be due to a low sensitivity of Northern blotting to detect CCR2 transcripts or cell culture conditions such as 2D culture system versus 3D culture system, hypoxic environment, and so on. Although it is unclear whether tumor cells of PDAC tissues have CCR2, presence of CCR2 was shown in preinvasive PanIN lesions of transgenic KC mice [13]. In addition, in a 3D culture system of murine duct-like cells derived from KC mouse pancreas, exogenous CCL2 treatment promoted cell proliferation through activation of ERK. These results suggested an autocrine mechanism of CCL2 used by pancreatic tumor cells to benefit their growth. Similar to pancreatic cancer, expressions of CCL2 and CCR2 were present in prostate cancer tissues and led to increased cancer proliferation and invasion through Akt signaling [82, 83]. Interference with this pathway by administration of CCR2 antagonist, CCL2 neutralizing antibody, or PI3 kinase inhibitor or by knockdown of CCL2 in prostate cancer PC3 cells all resulted in decreased tumor formation, smaller tumor size, and less metastases *in vivo* [82–84].

CXCR4 and its ligand CXCL12 were present in PanIN lesions and PDAC tissues and demonstrated to result in tumor proliferation, cancer progression, angiogenesis, and metastasis [85–87]. CXCR4-CXCL12-caused cell proliferation was mediated via EGFR-Src-PI3 kinase signaling pathway, which subsequently activates ERK [87–89]. For CXCR4-CXCL12-mediated invasion and metastasis of human pancreatic cancer cell lines, activation of Wnt, and Hedgehog signaling pathways were required and led to an EMT phenotype [90, 91]. Interestingly, inhibition of Wnt, Hedgehog, or CXCL12-CXCR4 signaling by pharmacological compounds resensitized cancer cells to gemcitabine-induced cytotoxicity, suggesting the importance of CXCL12-CXCR4 in PDAC drug resistance [92–94]. Similar to the functions of CXCR4-CXCL12 in PDAC, prostate cancer cells express high levels of CXCR4 and CXCL12 and utilize the same signaling pathways described above to modulate their proliferation, angiogenesis, drug resistance, and metastasis (see review in [95, 96]).

Cytokine granulocyte-macrophage colony-stimulating factor (GM-CSF) can affect tumor immune response positively or negatively depending on the local tumor microenvironment, which is distinct among cancer types [97]. Accumulating evidence supports a role of GM-CSF in promoting rather than inhibiting the development of pancreatic cancer. Higher expressions of GM-CSF were detected in human and mouse PDAC tissues and have been shown to modulate development of Gr-1^+^CD11b^+^ myeloid cells, which inactivate cytotoxic CD8^+^ T cells [98, 99]. In addition, blockade of GM-CSF derived from PDAC by either a GM-CSF-neutralizing antibody or a specific knockdown of GM-CSF in pancreatic cancer cells suppressed tumor growth with less infiltrating Gr-1^+^CD11b^+^ cells *in vivo*. Interestingly, GM-CSF secreted by cancer-associated mesenchymal stem cells (CA-MSC) was required for cell invasion, metastasis, and transendothelial migration of human pancreatic cancer [100]. In addition, it also modulated EMT and stemness of PDAC. In contrast, despite of unknown mechanism of how GM-CSF inhibits metastatic prostate cancer, GM-CSF has been in the clinical trials for prostate cancer patients since 2001. It has been shown that systemic GM-CSF given to the patients in the clinical trials altered immune cell populations with an increase in CD4^+^ T cells, CD8^+^ T cells, and mature myeloid dendritic cell and a decrease in Treg cells [101]. Interestingly, expressions of receptors for GM-CSF are reported in cultured human prostate carcinoma LNCaP cells [102].

Activation of Fas ligand (FasL) and its receptor (Fas) leads to cell apoptosis. In pancreatic cancer, expressions of FasL and Fas were present in human PDAC tissues and cultured cancer cell lines [103–105]. It has been demonstrated that FasL-expressed PDAC induced apoptosis of the infiltrating lymphoid cells, thus eliminating tumor-killing immune cells [103, 105]. Meanwhile, pancreatic cancer cells were resistant to Fas-induced apoptosis by downregulation of Fas or upregulation of Fas-associated phosphatase 1 (FAP-1), which is mediated by JNK and p38 MAPK [104]. Similar to the functions and expressions of FasL in pancreatic cancer, human prostate cancer cells were also resistant to FasL-induced apoptosis. In addition, soluble FasL (sFasL) was consistently secreted through a cleavage on membrane-bound FasL by MMPs in prostate cancer [106]. Tumor exosomes of human prostate carcinoma LNCaP cells expressed FasL and caused cytotoxic CD8^+^ T cell apoptosis [107]. It has been shown that interaction of FasL with Fas in the intracellular compartment resulted in cancer cell apoptosis of human prostate carcinoma cells that are resistant to anti-Fas antibody CH-11 [108].

## 5. Conclusion

Increased cytokine signaling in both tumor-infiltrating immune cells and cancer cells potentiates tumor growth, metastasis, and drug resistance of pancreatic and prostate cancers. Many cytokines upregulated in cancer cells are mediated through autocrine signaling. Meanwhile, cancer cells also use paracrine-signaling mechanism to recruit immune cells to the tumor site. These infiltrating immune cells then produce more cytokines to directly or indirectly support tumor growth. Certain downstream effectors of cytokines, especially upregulated by cancer cells, modulate stem cell-like properties such as Notch, Wnt, and Hedgehog. Through controlling these pathways, cancer cells are capable of proliferating and surviving even when under harsh conditions, for example, cancer therapy. A comprehensive view of this complex cytokine network among tumor cells, immune cells, and other types of cells including stromal cells, endothelial cells, and so on will provide invaluable information on best strategies to defeat cancer-caused death, for example, use of cytokine inhibitor cocktails in the future.
